# Neuronal Calcium Sensor Synaptotagmin-9 Is Not Involved in the Regulation of Glucose Homeostasis or Insulin Secretion

**DOI:** 10.1371/journal.pone.0015414

**Published:** 2010-11-09

**Authors:** Natalia Gustavsson, Xiaorui Wang, Yue Wang, Tingting Seah, Jun Xu, George K. Radda, Thomas C. Südhof, Weiping Han

**Affiliations:** 1 Laboratory of Metabolic Medicine, Singapore Bioimaging Consortium, Agency for Science Technology and Research (A*STAR), Singapore, Singapore; 2 Howard Hughes Medical Institute and Department of Molecular and Cellular Physiology, Stanford University School of Medicine, Palo Alto, California, United States of America; 3 Department of Biochemistry, Yong Loo Lin School of Medicine, National University of Singapore, Singapore, Singapore; University of Bremen, Germany

## Abstract

**Background:**

Insulin secretion is a complex and highly regulated process. It is well established that cytoplasmic calcium is a key regulator of insulin secretion, but how elevated intracellular calcium triggers insulin granule exocytosis remains unclear, and we have only begun to define the identities of proteins that are responsible for sensing calcium changes and for transmitting the calcium signal to release machineries. Synaptotagmins are primarily expressed in brain and endocrine cells and exhibit diverse calcium binding properties. Synaptotagmin-1, -2 and -9 are calcium sensors for fast neurotransmitter release in respective brain regions, while synaptotagmin-7 is a positive regulator of calcium-dependent insulin release. Unlike the three neuronal calcium sensors, whose deletion abolished fast neurotransmitter release, synaptotagmin-7 deletion resulted in only partial loss of calcium-dependent insulin secretion, thus suggesting that other calcium-sensors must participate in the regulation of insulin secretion. Of the other synaptotagmin isoforms that are present in pancreatic islets, the neuronal calcium sensor synaptotagmin-9 is expressed at the highest level after synaptotagmin-7.

**Methodology/Principal Findings:**

In this study we tested whether synaptotagmin-9 participates in the regulation of glucose-stimulated insulin release by using pancreas-specific synaptotagmin-9 knockout (p-S9X) mice. Deletion of synaptotagmin-9 in the pancreas resulted in no changes in glucose homeostasis or body weight. Glucose tolerance, and insulin secretion *in vivo* and from isolated islets were not affected in the p-S9X mice. Single-cell capacitance measurements showed no difference in insulin granule exocytosis between p-S9X and control mice.

**Conclusions:**

Thus, synaptotagmin-9, although a major calcium sensor in the brain, is not involved in the regulation of glucose-stimulated insulin release from pancreatic β-cells.

## Introduction

Maintenance of glucose homeostasis requires adequate amount and precise pattern of insulin secretion, which is tightly controlled and regulated in response to blood glucose levels and neuroendocrine cues [1], [2]. A failure in these mechanisms leads to hyperglycemia and type 2 diabetes, or life-threatening hypoglycemic conditions [1]. The basic cellular events in the excitation-secretion coupling of pancreatic β-cells are relatively well described, such as glucose uptake into β-cells and consequent changes in ATP/ADP ratio, membrane ion channel dynamics, and elevated cytoplasmic calcium as the trigger for insulin granule exocytosis [1], [2]. However, the precise mechanism and temporal events from calcium elevation to insulin granule exocytosis remain elusive, and we have just begun to define the proteins involved in calcium sensing in insulin secretion regulation [3], [4], [5].

Synaptotagmins, a family of at least 15 proteins, are primarily expressed in neurons and endocrine cells [4], [6]. Due to sequence variations at the Ca^2+^-coordinating sites and the flanking residues, only eight synaptotagmins bind Ca^2+^, and they exhibit diverse Ca^2+^ binding properties [4], [6]. Synaptotagmin-1, -2 and -9 regulate fast neurotransmitter release, and function as calcium sensors for neurotransmission of their respective brain regions [7], [8], [9]. These synaptotagmins have low calcium affinity and therefore can respond within milliseconds at the active zone where cytoplasmic calcium concentration is high (∼10–40 µM) to trigger synaptic vesicle (SV) exocytosis [4]. In contrast, exocytotic rate of insulin granules and of large-dense core vesicles (LDCV) in general, is usually slower, and requires lower calcium levels, although insulin granule exocytosis in β-cells and catecholamine release from chromaffin cells can also be triggered by high calcium concentrations [10], [11]. Despite the apparent differences between SV and LDCV exocytosis, the exocytotic machinery in neuronal and endocrine cells shows remarkable similarities [4], [12]. SNAREs operate during membrane fusion in both systems [12], [13] and synaptotagmins also function as calcium sensors in excitable endocrine cells [4], [14].

In our previous study, we identified synaptotagmin-7 as a high affinity calcium sensor in insulin secretion, and found that deletion of synaptotagmin-7 resulted in ∼40% reduction in calcium-dependent insulin release [15], which indicates that other proteins must be responsible for regulating the remaining 60% of insulin secretion. Besides synaptotagmin-7, several other calcium-binding synaptotagmin isoforms are present in pancreatic islets [15]. Of these, mRNA level of the neuronal calcium sensor synaptotagmin-9 is the highest after synaptotagmin-7 in pancreatic islets [15]. Since insulin release can be evoked by a wide range of calcium concentrations, from submicromolar to tens of µM [11], it is conceivable that the low affinity calcium sensor synaptotagmin-9 may regulate insulin secretion in response to high calcium concentrations, in collaboration with synaptotagmin-7, which is responsible for insulin release at low calcium concentrations.

To investigate whether synaptotagmin-9 functions as a calcium sensor in glucose-stimulated insulin release, we generated pancreas-specific synaptotagmin-9 KO (p-S9X) mice, and tested the effects of synaptotagmin-9 deletion on glucose homeostasis, and on insulin secretion *in vivo* and in pancreatic islets and β-cells.

## Methods

### Animal Welfare

All experiments involving animals were reviewed and approved by the Institutional Animal Care and Use Committee of A*STAR (Agency for Science, Technology and Research) under IACUC #080351 and #090428.

### Generation of Pancreas-Specific Synaptotagmin-9 KO Mice

Pancreas-specific synaptotagmin-9 KO mice (p-S9X) were generated by crossing synaptotagmin-9^fl/fl^ mice containing *Cre* transgene under pdx1 promoter (Pdx1-Cre) with synaptotagmin-9^fl/fl^ mice. The Pdx1-Cre mouse line was a generous gift from Dr. Doug Melton (Harvard University). Floxed synaptotagmin-9 (synaptotagmin-9^fl/fl^) mice were described previously [9]. Littermates with genotypes of synaptotagmin-9^fl/fl^/Pdx1-Cre (p-S9X) and synaptotagmin-9^fl/fl^ (control) were used in this study. Genotyping was performed on tail DNA by PCR using the following primers: 5′-TGC TTC TGT CCG TTT GCC GGT-3′ and 5′-CTA AGT GCC TTC TCT ACA CCT-3′ (PCR product of 500 bp indicates the presence of Cre transgene); 5′- GCG TGA GAA AAC TCA CTG GAT G-3′ and 5′- GTC CAC TAG GGC TAG CCC AGG-3′ (PCR fragment of 200 bp and 1100 bp indicate the presence of wild type and mutant allele, respectively); 5′- GAG AGT GAT AGA GAA AGA CAC-3′ and 5′- GTT GAG ATG TAA TGT ATA CCT ATG C-3′ (PCR product of 200 bp indicates the presence of mutant allele).

For islet isolation, histological staining, electron microscopy and qPCR, mice were sacrificed by cervical dislocation. Altogether, 60 control and 53 p-S9X mice were used. All mice used in this study were bred and housed in our animal facility.

### Measurements of Body Weight and Body Composition

Mice were fed *ad libitum* with free access to water. Body weight in p-S9X and control mice was monitored between 4 and 15 weeks of age. Body fat and lean mass was analyzed on 16 wk-old mice by using Echo MRI (Echo Medical Systems) as previously described [15].

### Glucose Tolerance Tests and Blood Glucose Measurements

Intraperitoneal glucose tolerance tests (IPGTT) was performed as previously described [15]. Briefly, 12–14 wk-old mice were fasted for 16–18 h with free access to water. Blood glucose levels were tested before and at 15, 30, 60, 90 and 120 min after i.p. injection of 20% D-glucose solution at 2 g/kg of body weight. ∼4 µl of tail vein blood was taken at each time point, and blood glucose levels were determined by using Accu-Chek Advantage blood glucose meter (Roche Diagnostics).

### Glucose-Stimulated Insulin Secretion *in vivo* and Plasma Insulin Measurements

Insulin secretion *in vivo* was induced by i.p. injection of 20% D-glucose solution at 2 g/kg of body weight on 12–14 wk-old mice after overnight fasting. ∼20 µl blood samples were collected from tail vein at 3, 8, 15, 30 and 60 min after glucose injection. Blood samples were mixed with 2 µl of 0.5 M EDTA on ice and centrifuged at 10,000× g for 5 min for plasma collection. Plasma insulin concentrations were determined by using Ultrasensitive Mouse Insulin ELISA Kit (Mercodia, Sweden).

### Histological Analysis and Electron Microscopy

For histological analysis, pancreata from 4 mice of each group (p-S9X and control mice) were fixed in 10% formalin at 4°C for 24 h before paraffin embedding. Then 5 µm serial sections from each sample were processed for routine histological study (hematoxylin-eosin). For transmission electron microscopy, pancreatic samples from 4 mice of each group (p-S9X and control mice) were fixed in 2.5% glutaraldehyde (Agar Scientific) and in 1% OsO_4_ in PBS (pH 7.4) at 4°C for 2 h each, dehydrated in ethanol series and embedded in araldite epoxy resin (EMS). Ultrathin (∼90 nm) sections were stained with 2% uranyl acetate (Analar) for 10 min and examined by using a JEOL JEM-1220 electron microscope (JEOL Asia Pte).

### Islet Isolation and Insulin Measurements

Islets from p-S9X and control mice were isolated by liberase digestion (0.25 mg/ml; Roche) and cultured overnight at 11.1 mM glucose in Advanced RPMI 1640 medium supplemented with 10% heat-inactivated fetal bovine serum, 1% penicillin/streptomycin, 2 mM GlutaMax and 15 mM Hepes, pH 7.4 (Invitrogen). Subsequent experimental handling was performed with a Krebs-Ringer-Hepes medium (KRH) containing (in mM) 130 NaCl, 4.7 KCl, 1.2 KH_2_PO_4_, 1.2 MgSO_4_, and 2.56 CaCl_2_, supplemented with 1 mg/ml BSA and 3 mM D-glucose. The medium was buffered with 20 mM Hepes and NaOH to pH 7.4 and equilibrated with ambient air. For estimation of islet insulin content, islets were lysed by sonication in 200 µl of passive lysis buffer (Promega) after incubation in KRH medium containing 3 mM glucose for 2 h. Insulin content in the buffer was measured by using Mouse Insulin ELISA (Mercodia).

For analysis of islet secretory responses, batches of 5 similar-sized islets from a single mouse were placed in a 40-µL flow chamber under continuous perifusion (0.3 ml/min) with KRH medium containing 3 mM glucose for 60 min at 37°C. The medium was then switched to KRH containing 20 mM glucose for 30 min. Fractions of the medium were collected every 3 min, starting at 6 min before stimulation. Insulin concentration in each fraction was measured by using Mouse Insulin ELISA (Mercodia).

### Ca^2+^ Measurements

Calcium measurements were performed as described previously [15]. Briefly, isolated and overnight-cultured islets were loaded with 3 µM Fluo-4 in KRH buffer containing 3 mM glucose for 1 hour, then placed in a perifusion chamber on the stage of an inverted microscope (Nikon Eclipse TE2000-U) and continuously superfused with KRH buffer containing 3 mM glucose at 37°C. Fluo-4 was excited at 488 nm by using a 175-watt xenon arc lamp, and emitted signal was projected onto a CCD camera (CoolSNAP HQ2, Photometrics) after passing through a 535DF35 band pass optical filter (Omega Optical). Images were collected every 2 sec, and fluorescence signals from individual cells were measured as a function of time using Metafluor software (Molecular devices). Fluo-4 images were acquired for 2 minutes in KRH buffer containing 3 mM glucose, and a further 10 minutes after the superfusion was switched to KRH buffer containing 20 mM glucose. Lag-time for [Ca^2+^]_i_ rise was defined as the time from the buffer switch (3 mM to 20 mM glucose) to the first value above baseline average, which was calculated during the 2 minutes before stimulation. Calcium rise was calculated as the difference between the average basal value and highest peak value.

### RNA Extraction and Quantitative RT-PCR

Total RNA was extracted from islets of p-S9X and control mice with TRIzol reagent (Invitrogen) according to the manufacturer's instructions. RNA samples were first treated with DNase I (Roche), and then reverse-transcribed with RevertAid (Fermentas). Quantitative real time PCR was performed by using SYBR Green chemistry and gene-specific primers on an Applied Biosystems StepOnePlus real-time PCR system. Primers used for detecting mouse synaptotagmin-9 (mus musculus synaptotagmin V) gene expression were designed by using PerkinElmer's Primer Express Software (5′-TAA GAC ACC TCC AGA CTC CA-3′ and 5′-TCA CTT CCT GAA GAT GGA CT-3′, accession number: NM_016908). Cyclophilin was used as the internal standard to determine relative mRNA levels between samples (5′-TGG AGA GCA CCA AGA CAG ACA-3′ and 5′-TGC CGG AGT CGA CAA TGA T-3′, accession number: NM_011149).

### Electrophysiology Measurements

Electrophysiology measurements were carried out essentially as previously described [16]. Briefly, Single β-cells were obtained by digestion of isolated pancreatic islets with 0.025 mg/ml trypsin [17], distributed on polylysine-coated cover glasses and cultured for 24 hours as described for islets. Membrane capacitance was recorded from single β-cells using standard whole-cell patch-clamp technique [18]. Exocytosis was elicited by a 500 ms depolarizing pulse from −70 to 0 mV. Single β-cells were identified by the absence of the transient Na^+^-current and the outward TEA-resistant K^+^-current when the cells were given a depolarization pulse from −70 to 0 mV. Pipette resistance ranged between 3 and 5 MΩ when pipettes were filled with intracellular solution containing (in mM): 135 KCl, 10 NaCl, 1 MgCl_2_, and 3 MgATP, 5 Hepes (pH 7.2 with KOH). Extracellular solution contained (in mM): 118 NaCl, 20 TEA-Cl (tetraethylammonium chloride), 5.6 KCl, 2.6 CaCl_2_, 1.2 MgCl_2_, 5 D-glucose, and 5 Hepes (pH 7.4, with NaOH). Cells were stimulated at low frequency (<0.05 Hz) to allow full recovery of exocytotic capacity between pulses. Measurements were performed by using EPC10 plus patch clamp amplifier and analyzed by using Patchmaster software. Exocytosis was detected as changes in cell membrane capacitance (Cm), which was estimated by the Lindau-Neher technique implementing the ‘Sine+DC’ feature of the lock-in module [19]. The amplitude of the sine wave was 10 mV and the frequency was set at 1 kHz. All Cm measurements were performed at 30–32°C.

### Statistical Analysis

Data are presented as means ± SEM. Comparisons of data from p-S9X and control mice were made by using Student's two-tailed t-test for independent data. The significance limit was set at P<0.05.

## Results

### Generation of pancreas-specific synaptotagmin-9 KO (p-S9X) mice

**Figure 1:**
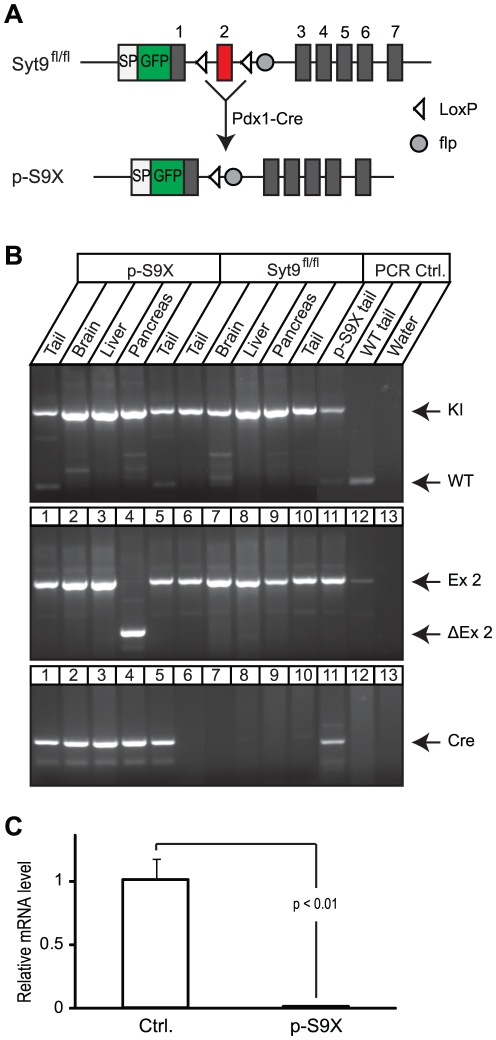
Generation of pancreas-specific synaptotagmin-9 KO (p-S9X) mice. (**A**) Homologous recombination and cre excision strategy for generating pancreas-specific synaptotagmin-9 KO mice. Syt9^fl/fl^ (synaptotagmin-9^fl/fl^) showing targeted exon 2 and flanking loxP sites, p-S9X showing synaptotagmin-9 gene structure after exon 2 deletion by cre recombination. (**B**) Representative genotyping patterns for p-S9X and Syt9^fl/fl^ mice. Note that deletion of exon 2 was only observed in the pancreas of p-S9X. (**C**) Synaptotagmin-9 mRNA levels were analyzed by quantitative real time PCR on total RNA extracted from p-S9X and Syt9^fl/fl^ (ctrl.) mouse islets. Data are presented as means ± SEM. N = 4 mice for each genotype. P<0.01.

To investigate whether synaptotagmin-9 was involved in the regulation of glucose homeostasis and insulin secretion, we generated pancreas-specific synaptotagmin-9 KO mice by crossing synaptotagmin-9^fl/fl^ with and without Pdx1-Cre transgene (Figure 1A). P-S9X and littermate synaptotagmin-9^fl/fl^ (control) mice were used in the experiments. Pdx1 promoter drove specific expression of Cre recombinase in the pancreas, with no detectable expression in other tissues, as further confirmed by the lack of Cre recombination activity outside the pancreas in p-S9X mice (Figure 1B). Under the Pdx1 promoter, Cre expression in the pancreatic epithelium started at early embryonic stages, which ensured efficient synaptotagmin-9 deletion in the pancreas (Figure 1C).

### Body weight, glucose and insulin levels

P-S9X mice were viable and fertile, and showed no differences from synaptotagmin-9^fl/fl^ in their postnatal development, including body weight gain and body composition (Figure S1A and S1B). We compared synaptotagmin-9^fl/fl^ with wild type mice, and found no difference in body weight, glucose tolerance and glucose levels (data not shown), therefore, we used synaptotagmin-9^fl/fl^ as control to p-S9X mice in subsequent experiments. P-S9X mice and their control showed similar fasting glucose levels (4.4±0.1 and 4.4±0.1 mmol/l, N = 27 and 25 for control and p-S9X, respectively; NS) and fed glucose levels (9.0±0.3 and 9.9±0.5 mmol/l, N = 13 and 15 for control and p-S9X, respectively; NS). Fasting insulin levels were the same (0.23±0.003 and 0.23±0.003 ng/ml, N = 21 and 23 for control and p-S9X, respectively; NS) and fed insulin levels were also similar in control and p-S9X mice (0.78±0.14 and 0.64±0.14 ng/ml, N = 8 and 7 for control and p-S9X, respectively; NS). These results indicate that pancreas-specific deletion of synaptotagmin-9 had no effect on glucose homeostasis in p-S9X mice.

### Glucose tolerance test and acute insulin response

**Figure 2:**
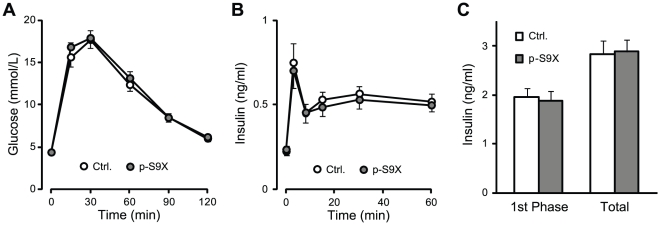
Normal glucose tolerance and *in vivo* insulin secretion in p-S9X mice. (**A**) Intraperitoneal glucose tolerance test (IPGTT) in p-S9X and control mice. p-S9X mice (filled circles, N = 16) showed the same glucose increase and clearance as control mice (open circles, N = 15). (**B**) Insulin secretion *in vivo* induced by i.p. injection of glucose in p-S9X and control mice. Plasma insulin levels were determined in control (open circle, N = 21) and p-S9X mice (filled circle, N = 21) after i.p. injection of glucose. (**C**) Area under curve for the insulin secretion curves in (B). Data are presented as means ± SEM. N = 21 for each genotype group.

Deletion of synaptotagmin-7, a calcium sensor in insulin secretion, resulted in glucose intolerance and impaired insulin secretion in synaptotagmin-7 KO mice, even though the KO mice exhibited normal resting and fed glucose levels [15]. To examine whether p-S9X mice were also defective in response to glucose overload, we performed glucose tolerance tests and measured glucose-induced insulin secretion *in vivo* in p-S9X and control mice. After i.p. glucose injection, blood glucose increase and clearance were similar in p-S9X and control mice (Figure 2A). Plasma insulin levels were determined at 3, 8, 15, 30 and 60 minutes after glucose injection. No difference was observed in glucose-induced insulin secretion *in vivo* (Figure 2B), and the area under curve for the first phase of insulin secretion or for the entire measurement period was the same in p-S9X and control mice (Figure 2C). These data indicate that glucose-stimulated insulin secretion in p-S9X mice was adequate to regulate glucose homeostasis under glucose overload, similar to that in control mice, and further support the notion that synaptotagmin-9 was not involved in the regulation of glucose homeostasis.

### Glucose-induced insulin release from isolated islets

**Figure 3:**
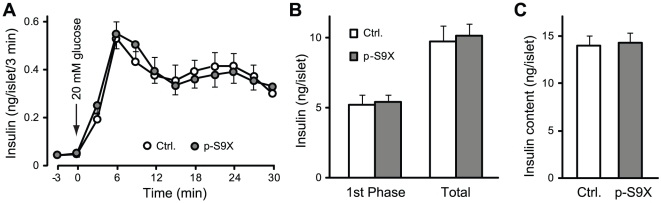
Glucose-stimulated insulin secretion from p-S9X mouse islets showed the same pattern as in control mouse islets. (**A**) Insulin secretion from batches of 5 similar-sized islets in perifusion experiments in response to 20 mM glucose (arrow). N = 20 for control mouse islets (open circles), and 16 for p-S9X mouse islets (filled circles). (**B**) Insulin secretion during the first 15 minutes of stimulation (first phase) and over the entire stimulation period (total), calculated as sum of insulin levels in all fractions after baseline subtraction. (C) Insulin contents in isolated individual pancreatic islets from p-S9X and control mice. Data are presented as means ± SEM. N = 14 for control (white bar) and 19 for p-S9X (grey bar).

The fact that p-S9X mice showed normal glucose tolerance and glucose-induced insulin secretion *in vivo* argues against synaptotagmin-9 as a calcium sensor in insulin granule exocytosis. Since synaptotagmin-9 was deleted during embryonic development, p-S9X mice might have developed mechanisms to compensate for defects in calcium-dependent insulin granule exocytosis, *e.g.* increased insulin content or increased number of islets in β-cells, and as such, we failed to detect impaired insulin secretion *in vivo* as measured in tail vein blood. To directly examine insulin secretion, we performed perifusion experiments on isolated pancreatic islets, and measured insulin secretion in response to glucose stimulation (Figure 3A). Basal insulin secretion at 3 mM glucose was the same in p-S9X and control mouse islets. High glucose challenge induced more than 15-fold enhancement in insulin secretion at 6 minutes in both p-S9X and control islets, representing the peak amplitude of the first phase of glucose-stimulated insulin secretion. Insulin levels in the fractions collected between 9 and 15 minutes gradually decreased, but continued to show sustained elevation above basal levels until the end of stimulation, corresponding to the second phase of insulin secretion. There was no difference in peak amplitude or time course of insulin release between p-S9X and control mouse islets. Consequently, net insulin secretion during the first 15 minutes of stimulation (first phase) and over the entire stimulation period was similar between p-S9X and control islets (Figure 3B). Islet insulin content was the same in control and p-S9X mouse islets (Figure 3C). These results showed that deletion of synaptotagmin-9 had no effects on the amount or kinetics of glucose-induced insulin secretion in p-S9X mice.

### Morphological and ultrastructural characterization

**Figure 4:**
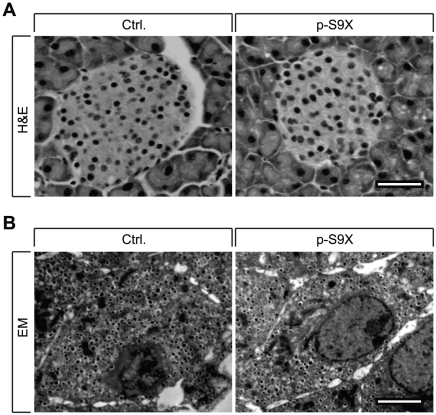
p-S9X mice exhibit similar islet architecture and β-cell ultrastructure as their control mice. (**A**) Histological sections stained with hematoxylin and eosin (H&E). Representative sections are shown for p-S9X and control islets. N = 4 mice for each genotype. Scale bar = 50 µm. (**B**) Transmission electron micrographs showing β-cells of p-S9X and control pancreas. Scale bars = 2 µm.

To examine the involvement of synaptotagmin-9 in the regulation of islet development and maintenance, we tested whether deletion of synaptotagmin-9 in the pancreas led to changes in islet architecture. Histological analysis of the pancreas showed no pathological signs in p-S9X mouse islets, and no differences with regard to the number, size or shape of islets between p-S9X and control mice (Figure 4A). Vascularization of islets and cell morphology were similar as well. We then examined pancreatic β-cell ultrastructure. Electron microscopy of pancreatic islets showed no differences between p-S9X and control mice in terms of the number and appearance of insulin granules and β-cell ultrastructure in general (Figure 4B). These data demonstrate that islet development and maintenance were not dependent on synaptotagmin-9, and that there were no compensatory changes in islet architecture or β-cell ultrastructure.

### Glucose-induced calcium responses

As calcium is the triggering signal for insulin granule exocytosis, alterations in glucose-induced calcium responses might have compensated for the deletion of synaptotagmin-9. To test this, we recorded intracellular calcium changes to high glucose stimulation in isolated islets from p-S9X and control mice. Glucose-induced Ca^2+^ responses showed a similar pattern in p-S9X and control mouse islets (Figure S2). Calcium rise (188±39 vs. 249±36 a.u. for control and p-S9X mouse islets, respectively; N = 23–26, NS) and lag time (88±7 vs. 74±7 s for control and p-S9X mouse islets, respectively; N = 23–26, NS) were the same for p-S9X and control mice.

### Insulin granule exocytosis in individual β-cells

**Figure 5:**
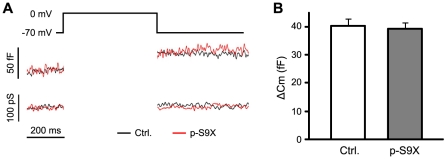
Normal membrane capacitance measurements in p-S9X mouse β-cells. (**A**) Membrane capacitance was recorded in functionally identified β-cells from p-S9X and control mice. Capacitance increase was elicited by 500 ms depolarizations from -70 to 0 mV. (**B**) Capacitance increases shown as means ± SEM. N = 7 for control mouse β-cells (white bar) and 9 for p-S9X (grey bar).

Considering that brain-specific deletion of synaptotagmin-9 resulted in specific loss of fast neurotransmitter release in neurons [9], and that synaptotagmin-9 may function as a low-affinity calcium sensor for fast insulin release, it is possible that we might have missed the insulin secretion defects in p-S9X mice in the perifusion experiments due to intrinsic limitation of low temporal resolution of this type of measurements. To better resolve calcium-dependent insulin release, we recorded capacitance changes in β-cells, as a measure of insulin granule exocytosis, in response to membrane depolarizations (Figure 5A). Isolated β-cells from p-S9X and control mice showed the same capacitance increases (Figure 5B), indicating that calcium-triggered insulin granule exocytosis was indistinguishable between p-S9X and control β-cells. These data exclude the involvement of synaptotagmin-9 in regulating insulin granule exocytosis.

## Discussion

Insulin, along with several other hormones and neuropeptides, is responsible for the proper maintenance of glucose homeostasis. Due to its great physiological effects in regulating glucose clearance from the blood, insulin secretion is tightly regulated. Insulin granule exocytosis can be triggered by a wide range of calcium concentrations [11]. Therefore calcium sensing in β-cells is expected to be complex, and may involve more than one calcium sensors with appropriate calcium affinity and cooperativity to respond to various intracellular calcium levels during a stimulus or under different stimuli. By analogy to neurotransmitter release, we hypothesized that insulin secretion is controlled by at least two types of calcium sensors: a low affinity sensor responsible for fast insulin release, and a high affinity sensor in charge of insulin release at low calcium concentrations.

We earlier identified synaptotagmin-7 as a high affinity calcium sensor for insulin secretion, whose deletion resulted in ∼40% reduction in insulin release [15]. In this study, we focused on synaptotagmin-9, the second most highly expressed calcium-binding synaptotagmin in pancreatic islets, and tested our hypothesis that synaptotagmin-9 functions as a low affinity calcium sensor in insulin granule exocytosis.

Pancreas-specific synaptotagmin-9 KO mice showed no differences in body weight, glucose tolerance, or glucose-induced insulin secretion *in vivo* and from isolated islets when compared to control mice. Deletion of synaptotagmin-9 also had no effects on pancreas development, islet insulin content, islet architecture or β-cell ultrastructure. Furthermore, calcium-triggered insulin granule exocytosis as detected by membrane capacitance measurements was normal in the absence of synaptotagmin-9. These results demonstrated that synaptotagmin-9 was not involved in the regulation of glucose homeostasis in general, or insulin secretion in particular. Although synaptotagmin-9 exhibited proper calcium binding properties required for insulin secretion, our study does not support our initial hypothesis that synaptotagmin-9 functions as a low-affinity calcium sensor in insulin granule exocytosis.

An earlier study showed insulin secretion was reduced by ∼30% in isolated rat islets with synaptotagmin-9 knockdown by adenoviral infection [20]. The apparent discrepancy between this and our present study may be due to our choice of the experimental subjects, as the pattern of glucose-induced insulin secretion is not identical in rat and mouse [21], [22]. It is possible that distinct patterns of calcium response in rat and mouse may result in different calcium levels, and thus requiring different calcium sensing proteins. The discrepancy could also be due to our choice in deleting synaptotagmin-9: genetic knockout vs. adenovirus-mediated RNAi, which may have undesired off-target effects [23].

The lack of effect by synaptotagmin-9 deletion in the pancreas could be the result of functional compensation by another synaptotagmin. We first generated synaptotagmin-7 and -9 double KO (DKO) mice, and compared insulin secretion in the DKO, synaptotagmin-7 KO and wild type control mice (refer to Materials and Methods S1). Although both DKO and synaptotagmin-7 KO mice showed impaired insulin secretion when compared with control mice, there was no further impairment in insulin secretion in DKO mice than in synaptotagmin-7 KO mice (Figure S3). In addition to synaptotagmin-7 and -9, which are expressed at high levels in pancreatic islets, two other calcium-binding synaptotagmins, synaptotagmin-3 and -5, are expressed at moderate levels in the islets [15]. We evaluated the expression of synaptotagmin-3, -5 and -7, but did not detect an upregulation of these synaptotagmins in p-S9X mice (data not shown). These results demonstrate that the lack of effect in insulin secretion by synaptotagmin-9 deletion was unlikely due to functional compensation, leading to the conclusion that synaptotagmin-9 is not a regulator of insulin secretion.

Besides synaptotagmins, other C_2_-containing proteins may participate in the regulation of insulin secretion. Previous studies have shown a potential role of the calpain family in insulin secretion [24], [25], possibly through partial proteolysis of SNAP-25 for the first phase [5] and ICA512 proteolysis for the second phase [26].

In conclusion, our results show that synaptotagmin-9 does not regulate glucose homeostasis or glucose-stimulated insulin secretion in mice. Our current efforts are focused on identifying the remaining calcium sensors for calcium-dependent insulin secretion beyond synaptotagmin-7.

## Supporting Information

Materials and Methods S1 (DOC)

Figure S1 **P-S9X mice exhibited similar body weight and body composition as their control mice.** (A) Body weight development of p-S9X and control mice. Body weights were measured on these two groups of mice between the age of 4 and 15 weeks. (B) Body fat and lean content in p-S9X and control mice were measured when the mice were 14–15 weeks old. Data are presented as means ± SEM. N = 9 for control (white bar) and 4 for p-S9X (grey bar). (EPS)

Figure S2 **Normal calcium response in p-S9X mouse islets.** Representative calcium responses induced by a rise in glucose concentration from 3 to 20 mM in control (red trace) and p-S9X (black trace) mouse islets (N = 23 and 26, respectively). P-S9X and control islets showed similar calcium response pattern with regard to timing of response (lag time) and amplitude of calcium rise (see RESULTS section for mean values and SEM). (EPS)

Figure S3 **Comparison of insulin secretion in control, synaptotagmin-7 KO and synaptotagmin-7/-9 DKO mouse islets.** Insulin secretion in response to 20 mM glucose was measured in batches of 5 similar-sized islets during the first 15 minutes (**A**, first phase) and the second 15 minutes (**B**, second phase). N = 18, 39 and 28 for control (Ctrl., white bar), synaptotagmin-7 KO (Syt7^−/−^, grey bar) and synaptotagmin-7/-9 DKO (DKO, black bar), respectively. Data are presented as means ± SEM. Statistics is indicated on the graph. NS: not significant. (EPS)

## References

[1] Henquin JC, Nenquin M, Ravier MA, Szollosi A (2009). Shortcomings of current models of glucose-induced insulin secretion.. Diabetes Obes Metab.

[2] Ashcroft FM, Proks P, Smith PA, Ammala C, Bokvist K (1994). Stimulus-secretion coupling in pancreatic beta cells.. J Cell Biochem.

[3] Gauthier BR, Wollheim CB (2008). Synaptotagmins bind calcium to release insulin.. Am J Physiol Endocrinol Metab.

[4] Gustavsson N, Han W (2009). Calcium-sensing beyond neurotransmitters: functions of synaptotagmins in neuroendocrine and endocrine secretion.. Biosci Rep.

[5] Marshall C, Hitman GA, Partridge CJ, Clark A, Ma H (2005). Evidence that an isoform of calpain-10 is a regulator of exocytosis in pancreatic beta-cells.. Mol Endocrinol.

[6] Südhof TC (2002). Synaptotagmins: why so many?. J Biol Chem.

[7] Geppert M, Goda Y, Hammer RE, Li C, Rosahl TW (1994). Synaptotagmin I: a major Ca2+ sensor for transmitter release at a central synapse.. Cell.

[8] Pang ZP, Melicoff E, Padgett D, Liu Y, Teich AF (2006). Synaptotagmin-2 is essential for survival and contributes to Ca2+ triggering of neurotransmitter release in central and neuromuscular synapses.. J Neurosci.

[9] Xu J, Mashimo T, Südhof TC (2007). Synaptotagmin-1, -2, and -9: Ca(2+) sensors for fast release that specify distinct presynaptic properties in subsets of neurons.. Neuron.

[10] Schonn JS, Maximov A, Lao Y, Südhof TC, Sorensen JB (2008). Synaptotagmin-1 and -7 are functionally overlapping Ca2+ sensors for exocytosis in adrenal chromaffin cells.. Proc Natl Acad Sci U S A.

[11] Barg S, Rorsman P (2004). Insulin secretion: a high-affinity Ca2+ sensor after all?. J Gen Physiol.

[12] Rettig J, Neher E (2002). Emerging roles of presynaptic proteins in Ca++-triggered exocytosis.. Science.

[13] Jahn R, Scheller RH (2006). SNAREs–engines for membrane fusion.. Nat Rev Mol Cell Biol.

[14] Gerber SH, Südhof TC (2002). Molecular determinants of regulated exocytosis.. Diabetes.

[15] Gustavsson N, Lao Y, Maximov A, Chuang JC, Kostromina E (2008). Impaired insulin secretion and glucose intolerance in synaptotagmin-7 null mutant mice.. Proc Natl Acad Sci U S A.

[16] Gustavsson N, Wei SH, Hoang DN, Lao Y, Zhang Q (2009). Synaptotagmin-7 is a principal Ca2+ sensor for Ca2+ -induced glucagon exocytosis in pancreas.. J Physiol.

[17] Lernmark A (1974). The preparation of, and studies on, free cell suspensions from mouse pancreatic islets.. Diabetologia.

[18] Gopel S, Zhang Q, Eliasson L, Ma XS, Galvanovskis J (2004). Capacitance measurements of exocytosis in mouse pancreatic alpha-, beta- and delta-cells within intact islets of Langerhans.. J Physiol.

[19] Lindau M, Neher E (1988). Patch-clamp techniques for time-resolved capacitance measurements in single cells.. Pflugers Arch.

[20] Iezzi M, Eliasson L, Fukuda M, Wollheim CB (2005). Adenovirus-mediated silencing of synaptotagmin 9 inhibits Ca2+-dependent insulin secretion in islets.. FEBS Lett.

[21] Zawalich WS, Zawalich KC (1996). Species differences in the induction of time-dependent potentiation of insulin secretion.. Endocrinology.

[22] Zawalich WS, Yamazaki H, Zawalich KC (2008). Biphasic insulin secretion from freshly isolated or cultured, perifused rodent islets: comparative studies with rats and mice.. Metabolism.

[23] Svoboda P (2007). Off-targeting and other non-specific effects of RNAi experiments in mammalian cells.. Curr Opin Mol Ther.

[24] Turner MD, Fulcher FK, Jones CV, Smith BT, Aganna E (2007). Calpain facilitates actin reorganization during glucose-stimulated insulin secretion.. Biochem Biophys Res Commun.

[25] Turner MD (2007). Coordinated control of both insulin secretion and insulin action through calpain-10-mediated regulation of exocytosis?. Mol Genet Metab.

[26] Ort T, Voronov S, Guo J, Zawalich K, Froehner SC (2001). Dephosphorylation of beta2-syntrophin and Ca2+/mu-calpain-mediated cleavage of ICA512 upon stimulation of insulin secretion.. EMBO J.

