# Supplementary material for: Neuronal Calcium Sensor Synaptotagmin-9 Is Not Involved in the Regulation of Glucose Homeostasis or Insulin Secretion
Source: PLoS One. 2010 Nov 9;5(11):e15414. doi: 10.1371/journal.pone.0015414 (PMC2976867; doi:10.1371/journal.pone.0015414)
Supplement: Materials and Methods S1 — (DOC) [file pone.0015414.s001.doc]

**SUPPLEMENTAL MATERIALS for Gustavsson *et al.***

**Neuronal calcium sensor synaptotagmin-9 is not involved in the regulation of glucose homeostasis or insulin secretion**

**MATERIALS AND METHODS:**

**Generation of synaptotagmin-7/-9 double KO mice:**

Synaptotagmin-7/-9 double KO (DKO) mice were generated by crossing synaptotagmin-9^fl/fl^ mice with and without pdx-cre on the background of synaptotagmin-7 KO.

**Insulin secretion measurements of islets from synaptotagmin-7/-9 DKO, synaptotagmin-7 KO and wt control mice:**

Batches of 5 isolated islets of similar size were incubated in 200 µl of KRH containing 3 mM glucose for 15 minutes for basal secretion. The medium was then collected and changed to KRH containing 20 mM of glucose for 15 minutes. After that the medium was replaced with new 20 mM glucose medium and incubated for another 15 minutes. All experiments were performed at 37 °C. Insulin concentration was measured in the collected basal, first phase and second phase fractions as described in the main text. 6-8 mice of each genotype were used.
